# Nanocomposites for Multifunctional Sensors: A Comprehensive Bibliometric Exploration

**DOI:** 10.3390/nano15010034

**Published:** 2024-12-29

**Authors:** Antonio del Bosque, Georgios Lampropoulos, Diego Vergara

**Affiliations:** 1 Technology, Instruction and Design in Engineering and Education Research Group (TiDEE.rg), Catholic University of Ávila, C/Canteros s/n, 05005 Ávila, Spain; 2 Department of Applied Informatics, University of Macedonia, 54636 Thessaloniki, Greece; glampropoulos@uom.edu.gr; 3 Department of Education, University of Nicosia, 2417 Nicosia, Cyprus

**Keywords:** multifunctional, nanocomposites, sensor, bibliometric analysis, review

## Abstract

Multifunctional nanocomposites have become critical components in advancing sensing technologies, owing to their exceptional integration of mechanical, electrical, thermal, and optical properties. The research landscape of nanocomposites for sensing applications from 2002 to 2024 is examined in this bibliometric review. It identifies key trends, influential works, prominent research areas, and global collaboration networks. This study highlights the creative significance of materials like metal–organic frameworks, carbon-based nanocomposites, and MXenes, which have been instrumental in advances, especially in hybrid systems that improve robustness and sensitivity. Offering an in-depth perspective on current research directions and emerging topics, this review explores areas like eco-friendly nanocomposites and additive manufacturing. Highlighting the relevance of biodegradable materials in supporting global sustainability efforts, it provides insights into future opportunities for advancing multifunctional nanocomposites in sensing technologies.

## 1. Introduction

In recent years, materials science has made substantial efforts in developing multifunctional nanocomposites. These materials, designed to perform multiple tasks by precisely combining structural and non-structural functions, go beyond conventional materials, which are typically engineered for a single primary function (such as mechanical strength or thermal conductivity) [1,2]. Multifunctional nanocomposites integrate a range of properties—mechanical, electrical, thermal, optical, and chemical—that empower them to perform complex functions [3]. By incorporating nanoscale conductive components like carbon or metal nanoparticles, researchers have engineered nanocomposites with outstanding electrical, mechanical, and optical properties [4].

Regarding nanocomposites for sensing applications, recent interest has focused on conductive nanoparticle-based polymers. Most of these types of sensors are based on a piezoresistive effect. The integration of conductive nanoparticles into otherwise insulating polymer matrices enables the formation of percolating networks, which significantly enhance electrical conductivity once a critical concentration, known as the percolation threshold, is reached—a phenomenon that is well documented in the field [5,6]. Carbon nanoparticles are favored over metallic alternatives due to their lower cost and proven sensitivity [7,8]. The electrical behavior of these types of nanocomposites is influenced by three primary factors: the intrinsic resistivity of the nanoparticles, contact resistance between adjacent particles [9], and the tunneling effect that occurs between neighboring particles [10]. When these materials are subjected to some condition that manifests mechanical strain (gas, mechanical strain, humidity, pressure, etc.), the electrical network is altered, leading to notable changes in conductivity. By tracking these electrical changes, it is possible to gain valuable insights into the materials’ strain state, making them ideal for advanced sensing applications. As commented before, extensive research over the years has focused on developing nanocomposites for monitoring applications. However, recent advancements are adding new functionalities to these nanocomposites for sensing, leading to the emergence of multifunctional nanocomposites for sensing technologies.

The versatility of multifunctional nanocomposites has led to their adoption across a wide range of sensing applications. In environmental monitoring, these materials have proven invaluable for detecting pollutants, heavy metals, and toxic gases with high precision [11,12]. The biomedical field has benefited greatly from nanocomposite-based biosensors, which offer improved methods for disease marker detection and physiological monitoring [13,14,15,16]. In the realm of structural health monitoring, nanocomposites have enabled the development of advanced strain and damage sensors for engineering structures [17,18]. Additionally, the food industry has embraced these materials for enhancing food safety through the detection of contaminants and quality control measures [19,20]. The security and defense sectors have also recognized the potential of nanocomposites in creating more effective sensors for chemical and biological threat detection [21,22].

**Figure 1:**
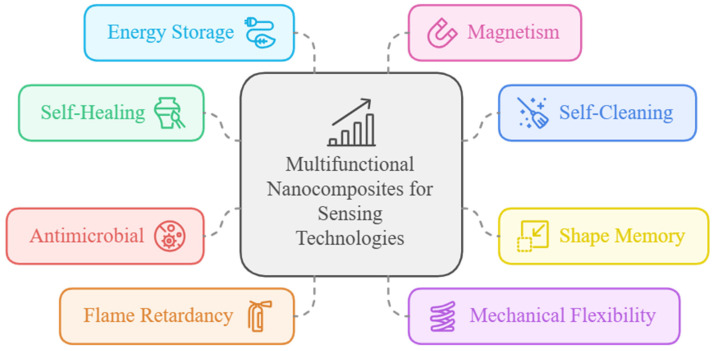
Applications of multifunctional nanocomposites for sensing technologies.

Multifunctional materials with integrated sensing capabilities are increasingly designed to include additional properties that enhance their performance and broaden their applications (Figure 1). Self-healing abilities, for instance, allow materials to repair themselves after minor damage, maintaining structural integrity and functionality over time—a critical feature for sensors in demanding or inaccessible environments [23]. Self-cleaning or self-washable properties, which can include hydrophobic surfaces or photocatalytic coatings, help sensors repel contaminants or degraded pollutants, thereby ensuring accurate measurements and reducing maintenance [24]. Shape memory is another valuable function, allowing materials to return to a preset shape after deformation, which is especially useful in dynamic or responsive systems where repositioning or adaptation is required [25].

Antimicrobial properties protect against bacterial or fungal growth, which is essential in medical or food industry applications where sterility and longevity are paramount [26,27]. Flame retardancy further extends the use of multifunctional materials in safety-critical environments by reducing the risk of fire, making them viable for applications in hazardous settings [28]. Mechanical flexibility, another key feature, allows materials to bend, stretch, or conform to irregular surfaces, ideal for wearable sensors or flexible electronics that require robustness and adaptability [29]. Moreover, some multifunctional materials incorporate energy storage capabilities, enabling them to store and supply energy, which can support low-power sensing devices [30]. Finally, magnetism can be integrated to allow for remote actuation or positioning, opening potential applications in data storage, biomedical targeting, or responsive systems [31]. These combined functionalities make multifunctional materials resilient and highly adaptable, enabling them to meet the complex and diverse demands of modern technological applications.

One of the most significant advantages of multifunctional nanocomposites in sensing technologies is their ability to be tailored for specific applications. By carefully selecting the nanomaterials and polymer matrices, researchers can design nanocomposites with properties optimized for sensing tasks [32]. This customization extends to the physical form of the sensors, allowing for the creation of flexible, wearable, and even implantable devices [33]. Such versatility opens new possibilities for real-time and continuous monitoring in diverse environments, from industrial settings to the human body. The adaptability of these materials has been a key factor in their rapid adoption and continued development across various scientific and technological domains.

The integration of artificial intelligence (AI) in the development of multifunctional nanocomposites has revolutionized the design and functionality of these materials, enabling the optimization of their properties and adaptation to complex applications, particularly in sensing technologies. AI facilitates the analysis of large volumes of experimental and theoretical data, allowing for predictions of behaviors and the customization of nanocomposite properties to meet specific needs. Machine learning enhances the exploration of material combinations for sensitive environmental sensors [34], while AI improves synthesis efficiency and property prediction for biomedical nanocomposites [35]. Additionally, AI optimizes durability and resistance, vital for creating resilient, reliable sensors in harsh conditions [36].

Despite the availability of reviews on nanocomposites for sensing technologies [8,37,38], few have utilized bibliometric tools to systematically analyze the research field. This bibliometric review analyzes the research landscape of nanocomposites in sensing applications, identifying key trends, influential publications, primary research areas, and collaborative networks. For this purpose, this paper aims to answer the following questions: What are the emerging trends and research hotspots in multifunctional nanocomposites for sensing technologies? Which materials and applications have received the most attention, and what are their limitations? How can collaborative networks and publication patterns inform future research directions? This review offers a comprehensive overview of current research directions and emerging themes, highlighting both the innovations and challenges shaping the future of multifunctional nanocomposites in sensing technologies.

## 2. Methods

**Figure 2:**
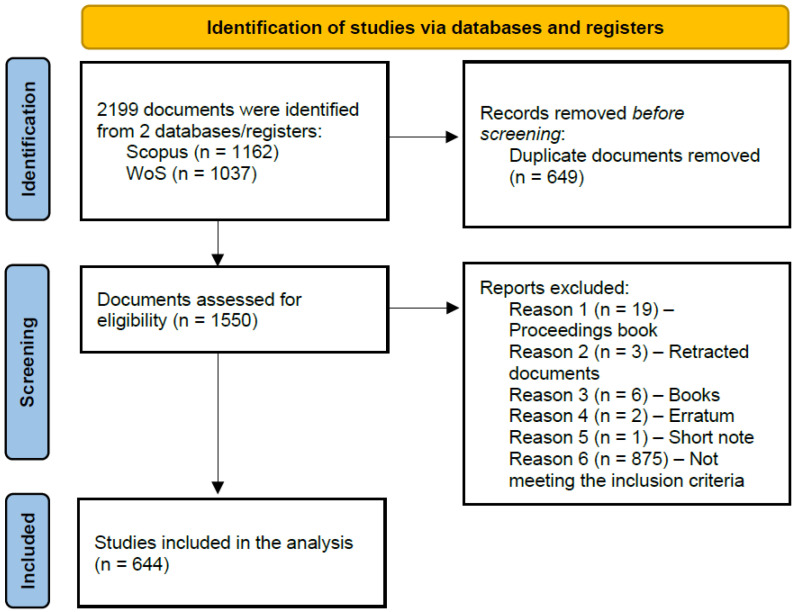
Comprehensive workflow for document processing.

To carry out this study, the PRISMA 2020 framework was adopted [39], and the Bibliometrix tool was used [40]. Each of the document processing steps is presented in Figure 2. Specifically, in October 2024, the following query was used to search for relevant data within the Scopus and Web of Science (WoS) databases: ““multifunctional” AND (“nanocomposite” OR “nanocomposites”) AND (“sensor” OR “sensing”)”. The specific databases were selected to their rigor and their being widely used to carry out review studies. Another reason for their selection was their ability to work well with our selected tool, Bibliometrix. Initially, 2199 English documents were identified out of which 1162 derived from Scopus and 1037 from WoS. A total of 649 documents were removed due to their being duplicates, and as a result, 1550 documents were assessed for eligibility. To include all possible related documents, a more generic inclusion criterion was set, and that was for the study to directly involve and combine the topics of multifunctionality, nanocomposites, and sensing. Thus, when processing the documents, 875 were removed as they did not meet the inclusion criteria, and 31 were removed for different reasons relevant to the information retrieved and their type which are presented in Figure 2. As a result, the final data collection examined in this study involved 644 documents published from 2002 to October 2024.

## 3. Results

The Results section presents a detailed analysis of the research landscape on multifunctional nanocomposites for sensing technologies, highlighting trends in publication growth, prominent materials, and emerging themes. This section aims to provide a comprehensive view of the field’s evolution while connecting diverse research directions and illustrating the global impact of advancements in sensing materials.

The document collection created was examined using the Bibliometrix tool to identify key aspects contained within the documents. Emphasis was placed on the statistics of the document collection, the annual citations and publications, the sources used to publish the related documents, as well as the affiliations and countries of the authors. The documents were further analyzed to identify the total citations per document and the keywords used as well as to explore the trend topics and conceptual structure of the use of multifunctional nanocomposites for sensing technologies.

### 3.1. Document Collection

**Figure 3:**
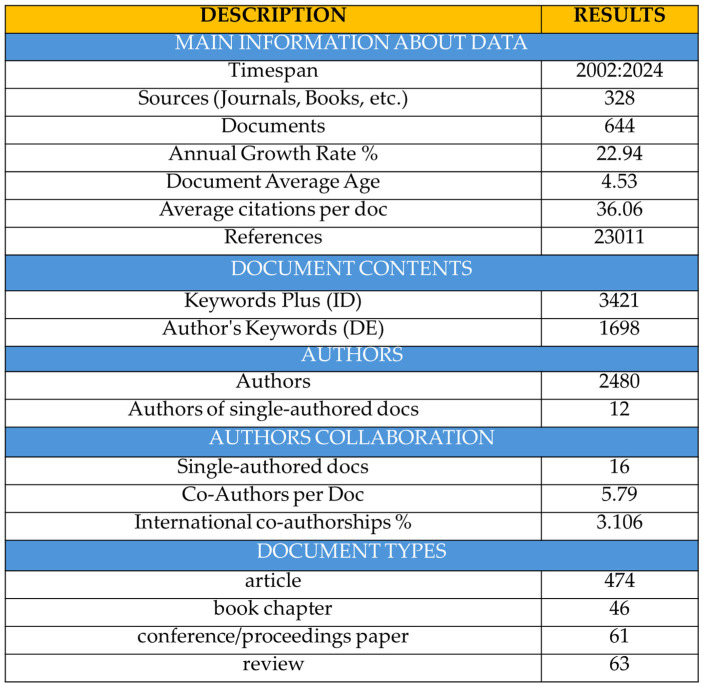
Document collection information.

Figure 3 summarizes the information relevant to the document collection examined in this article. During the period 2002–2024, a total of 644 documents were published in 328 different sources. Most of the documents were published as articles (73.6%), followed by review studies (9.8%) and conference papers (9.5%). A total of 2480 authors have contributed to the creation and publication of these documents out of which only 16 were single authored. Additionally, although there was an average of 5.79 co-authors per document, the international co-authorship rate was extremely low (3.1%). The documents had an annual growth rate of 22.94%, an average age of 4.53 years, and received 36.06 citations on average.

### 3.2. Annual Published Documents and Citations

**Figure 4:**
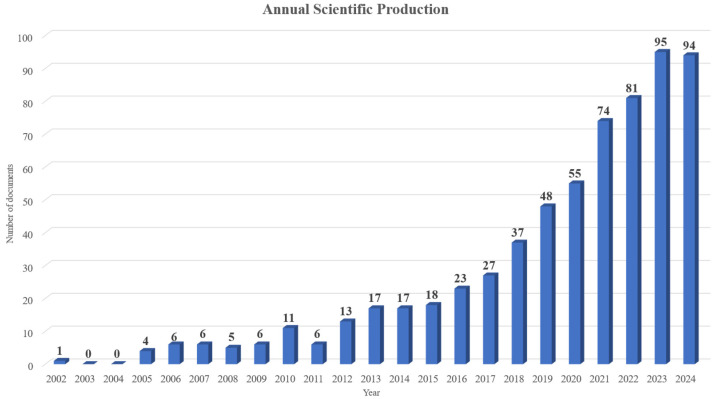
Annual documents published (2002–2024 October).

Having a high annual growth rate (22.94%) and viewing the results presented in Figure 4, it is obvious that the vast majority of studies were published in the last few years. Specifically, the years 2023 and 2024 have the most published documents, with over 90 documents published. Based on Figure 4, the following publication periods can be determined.

The first period (2002–2012) was characterized by sporadic scientific activity, with minimal annual publications. This phase likely focused on exploratory research, establishing the foundational properties of multifunctional nanocomposites without specific applications in sensing technologies. The second period (2013–2017) saw a gradual increase in publications, reflecting a growing interest in these materials’ potential for sensor applications, with studies focusing on material optimization. The third period (2018–2020) marked a more rapid growth, with 30–50 publications per year, indicating that nanocomposites were being tested in practical sensing applications. Finally, the fourth period (2021–2024) shows an exponential rise in research output, with over 90 documents published annually in 2023 and 2024, underscoring the field’s maturation and the increasing demand for advanced sensing technologies.

Table 1 reveals that the documents with the highest mean citation counts per article were published in 2015 (112.94 citations) and 2014 (103.71 citations), underscoring the influential nature of research from these years. Although these publications represent a smaller proportion of total output, they have garnered significant academic attention, likely due to pioneering findings or comprehensive reviews that provided critical insights or catalyzed new directions in multifunctional nanocomposites for sensing technologies. The high citation rates for these documents also reflect the impact of longevity in academic citation patterns; older publications naturally accrue more references over time, enhancing their visibility and authority in the field. The influence of studies from 2013 to 2015 is particularly noteworthy, as they appear to have shaped the trajectory of subsequent research, with their foundational methodologies and discoveries guiding modern advancements. This period has thus left a legacy on the field’s growth, with these highly cited documents continuing to be essential references in ongoing investigations, underscoring the pivotal role that early contributions play in shaping and sustaining the momentum of emerging fields.

### 3.3. Sources

Table 2 presents the most impactful journals based on the h-index, g-index, m-index, and total citations (TCs). The h-index measures both productivity and impact by counting the number of publications (h) that have received at least h citations each; for instance, an h-index of 19 means 19 publications have at least 19 citations each. The g-index emphasizes highly cited publications by considering the cumulative distribution of citations, giving more weight to influential articles; a g-index higher than the h-index indicates a concentration of citations in a few key works. The m-index normalizes the h-index by dividing it by the number of years of academic activity, reflecting the impact per unit of time. The journals *ACS Applied Materials and Interfaces* and *ACS Nano* emerge as the most influential sources in this domain, exhibiting the highest h-index values of 19 and 12, respectively. Particularly noteworthy, *ACS Nano* stands out due to the significant volume of citations it has garnered, totaling 3559 citations, nearly double that of the next-highest-cited source. These metrics indicate that these two journals are not only prolific in terms of the number of high-impact articles but also highly cited, reflecting their central role in disseminating research on nanocomposites for sensing applications. Other prominent journals, such as *Advanced Functional Materials* and *Nanoscale*, also display a notable h-index and citation figures, further solidifying their positions as key contributors to the literature.

In addition to analyzing citation-based impact, Bradford’s law was utilized to classify journals based on their document output in this field. As shown in Table 3, the sources were organized into three clusters. Cluster 1, which includes the journals with the highest document frequency, consists of the top 20 journals contributing a total of 213 articles. Among them, *ACS Applied Materials and Interfaces*, *ACS Nano*, and *Chemical Engineering Journal* rank as the most productive sources. Cluster 2 encompasses 54 journals, which collectively published 219 documents, while Cluster 3 comprises 62 journals with 212 publications. This clustering indicates that while a select few journals (Cluster 1) dominate the field in terms of productivity and impact, a broad array of sources also contributes to the diverse research landscape, highlighting the interdisciplinary nature of nanocomposite research for sensing technologies.

### 3.4. Affiliations and Countries

**Figure 5:**
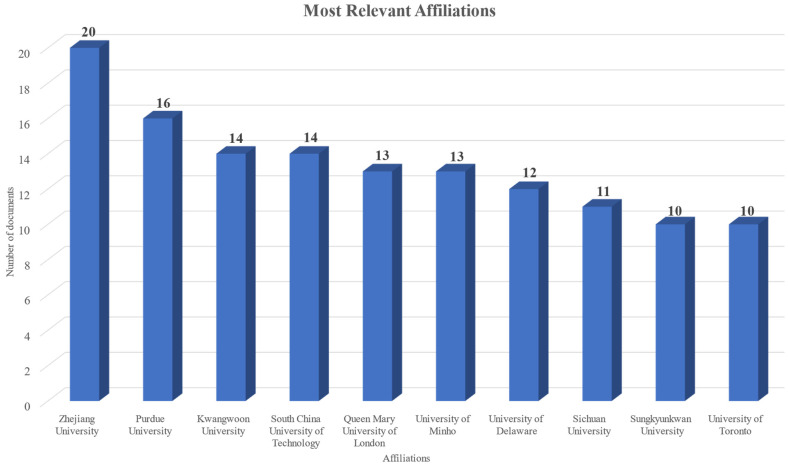
Top affiliations based on the number of documents published.

To determine the institutes that mostly focused on this topic, the total number of published documents according to the corresponding author’s or the first author’s affiliation were examined. Figure 5 presents the related outcomes based on which it becomes evident that “Zhejiang University”, “Purdue University”, “Kwangwoon University”, and “South China University of Technology” were the affiliations that had the most published documents in this area.

Additionally, using the same criteria to determine the country, Table 4 presents the countries whose authors published the most documents on the topic. Based on the results, China was the country whose authors predominantly focused on exploring this field. Specifically, the number of documents is more than double than that of the following two countries, that is India and the United States. This distribution reflects China’s active involvement and substantial resources allocated to advancing sensor technology using nanocomposites, positioning it as a leading contributor to this rapidly evolving research domain. The high number of intra-country collaborations (SCP) and, simultaneously, the lack of inter-country collaborations (MCP), as it becomes more evident from the data presented in Table 4, further highlights the lack of international collaborations which was also evident from the low international co-authorship rate.

**Figure 6:**
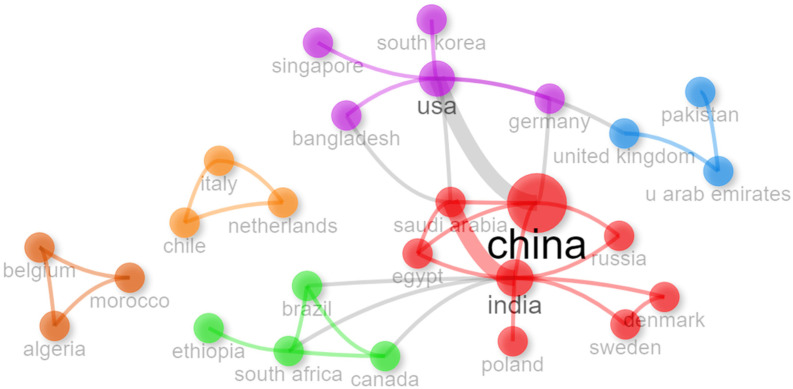
Country collaboration network.

The international collaborations in multifunctional nanocomposites for sensing technologies are shown in Figure 6, which reveals six main clusters. The largest cluster centers around China, highlighting strong connections with India, Saudi Arabia, and the United States, emphasizing China’s leadership and extensive network in this research area. Other key clusters include European countries like Italy, the Netherlands, and Germany, which form a robust regional network, and the United States with partners such as South Korea and Singapore, showcasing transcontinental collaborations. Smaller clusters, including countries in South America and Africa, indicate emerging partnerships, underscoring the global and interdisciplinary nature of this field.

The total citations received per country were also investigated. The related results are presented in Table 5. Specifically, it is evident that China, which had most of the published documents, received the highest number of total citations with a significant difference between the countries that followed, such as the United States, India, Japan, South Korea, and Canada. However, it is worth noting that documents from Japan received the highest number of citations per document on average.

### 3.5. Document Analysis

Using the total citations received within the documents of this collection, the most impactful documents were revealed. Specifically, Table 6 presents the top 11 documents according to the total citations they received. Based on the outcomes, the studies of Kaneti et al. [41], Zhao et al. [42], Quin et al. [43], Lin et al. [44], and Wang et al. [45] were the top 5 that received the most citations. Additionally, the study of Ma et al. [46] had the most total citations per year and the highest normalized total citations.

In summary, the Results section demonstrates the rapid evolution of research on multifunctional nanocomposites for sensing technologies, with a marked increase in publications and citations over the last decade. Key trends include the dominance of carbon-based nanocomposites, the emergence of MXenes and metal–organic frameworks, and significant advancements in hybrid materials. These findings underscore the field’s interdisciplinary nature, driven by the demand for innovative materials with high sensitivity, flexibility, and sustainability. Furthermore, the data reveal the importance of international collaborations and the growing role of additive manufacturing in shaping future research directions.

## 4. Discussion

In this section, we provide an in-depth analysis of the primary research themes identified through our bibliometric evaluation, highlighting the substantial growth of research on multifunctional nanocomposites for sensing technologies in recent years. The discussion focuses on the most impactful trends, key words, key authors, and international collaboration patterns that collectively shape the current landscape of nanocomposite research for sensing applications.

### 4.1. Document Trends

The most impactful documents shown in Table 6 provide a comprehensive basis for understanding how these materials advance towards integrating unique functionalities for different applications. These papers can be grouped into three key areas in multifunctional sensing technologies: (i) porous materials and hybrid structures for optimizing conductivity and sensitivity, (ii) developments in flexible and self-healing materials for real-time monitoring and detection applications, and (iii) sustainable and biodegradable nanocomposites in sensor technologies.

The first group is related to the use of porous materials such as metal–organic frameworks and hybrid structures with MXenes because they represent a strong research trend in nanocomposites for sensors. The work of Kaneti et al. highlights how MOFs, with their tunable structural and compositional characteristics, are ideal for sensing applications due to their high surface area and controllable porosity [41]. These materials not only enable efficient molecular signal capture but also support integration with other materials to form hybrid structures. Meanwhile, Zhao et al. introduces MXenes as key components in the construction of hybrid graphene nanocomposites, which significantly enhance electrical conductivity and are useful in applications such as electromagnetic interference shielding [42]. Both studies agree that combining MOFs and MXenes allows for the creation of sensors with high sensitivity and stability, particularly in extreme environments and applications requiring rapid and precise electromagnetic responses.

A second important trend is observed in the development of flexible and self-healing nanocomposites, designed for real-time monitoring and detection on dynamic surfaces such as human skin. Studies like that of Wang et al., which explores the flexibility of graphene oxide- and polyimide-based structures, highlight how these nanocomposites offer superelasticity and high recovery capacity, essential for wearable devices that must withstand repeated deformation cycles without performance loss [45]. These applications extend to biomedical sensors that monitor muscle movement and other physical parameters in real time. Similarly, Li et al. introduce MXene-based hydrogels, which possess remarkable self-healing capabilities and are degradable, making them suitable for temporary sensor applications in physiological monitoring and human–machine interactions [50]. Both studies emphasize the importance of integrating flexibility, high sensitivity, and mechanical strength into sensors so they can operate continuously without significant wear, confirming the viability of advanced hydrogels in portable, autonomous medical devices.

The last important trend is related to sustainability and biodegradability, which have become key goals in nanocomposite research for sensors, driving the development of materials based on natural resources and easy disposability. In this context, the study by Golmohammadi et al. underscores the potential of nanocellulose as an emerging platform in sensor and biosensor technologies [49]. Nanocellulose, a renewable nanomaterial, is valued not only for its exceptional mechanical and optical properties but also for its biodegradability, allowing its application in disposable sensors for medical diagnostics, environmental monitoring, and food quality control. Similarly, hydrogels studied by Wang et al. are presented as self-adhesive, self-healing materials that are highly sensitive to deformation [48]. These properties make hydrogels suitable for skin sensors in biomedical and sports applications, offering an option that minimizes environmental impact by reducing the use of plastics and non-biodegradable components.

In these works, we can perceive that carbon-based nanocomposite, MOFs, and MXenes are pivotal in advancing sensing technologies due to their complementary properties (Table 7). Carbon-based nanocomposites, such as graphene and carbon nanotubes, offer exceptional electrical conductivity, mechanical strength, and chemical stability. However, their performance can be further enhanced through the integration of other nanomaterials. MXenes, in comparison, provide unique advantages over traditional carbon additives, including higher electrical conductivity and tunable surface chemistry which facilitate better dispersion in polymer matrices. These attributes also allow MXenes to form strong interfacial interactions with other materials, leading to improved mechanical and functional properties. On the other hand, the availability of diverse dispersed fillers stems from the need to tailor material properties for specific sensing applications. Each filler offers unique benefits—carbon nanotubes provide excellent flexibility, graphene ensures high surface area and conductivity, and MOFs contribute tunable porosity and chemical functionality. By combining these materials, researchers can address limitations inherent to individual fillers, such as agglomeration or limited chemical specificity.

Finally, the use of hybrid structures is driven by the demand for multifunctionality in modern sensors. Hybridization leverages the strengths of individual components to achieve synergies that enhance overall performance. For example, combining MOFs with MXenes or carbon-based nanomaterials results in sensors with superior sensitivity, robustness, and environmental stability. Such hybrid systems also open opportunities for applications requiring simultaneous electrical, thermal, and mechanical optimization.

### 4.2. Keywords Trends

**Figure 7:**
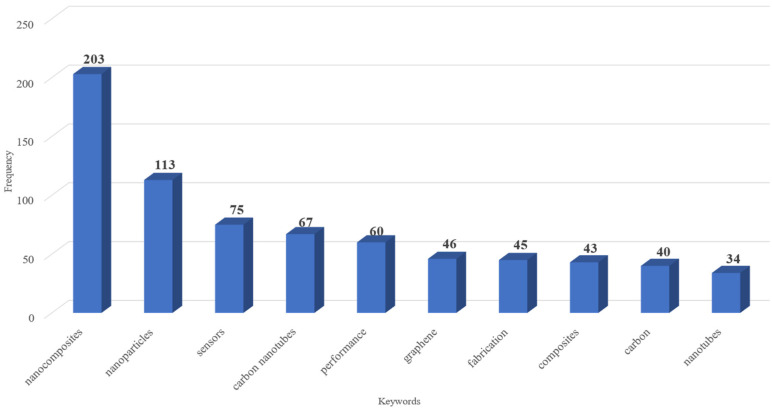
Most frequent keywords.

**Figure 8:**
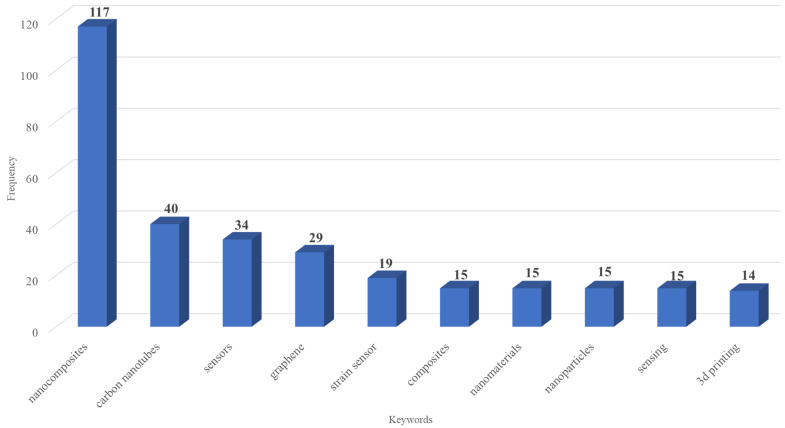
Most frequent author’s keywords.

The analysis of keywords (Figure 7 and Figure 8) reveals key areas of focus in the research on nanocomposites for sensing technologies.

Figure 7 shows the most frequent keywords as a set of terms added by databases to capture additional context from titles and abstracts. This selection highlights a strong emphasis on carbon-based nanocomposites (e.g., carbon nanotubes and graphene) and properties (e.g., performance and fabrication techniques), reflecting ongoing efforts to improve sensor functionality through advanced material engineering.

On the one hand, carbon nanoparticles (carbon nanotubes, graphene, nanofibers, and carbon black) possess remarkable properties that make them highly suitable for sensing applications [52,53,54,55]. These properties include excellent electrical conductivity and high surface area, mechanical strength, and chemical stability, which are crucial for achieving sensitivity and selectivity in sensing technologies [23]. These characteristics allow carbon-based nanocomposites to detect minute changes in environmental parameters, making them effective in applications such as gas sensors, biosensors, and wearable devices for health monitoring [56,57]. For instance, carbon nanocomposites have been effectively employed in strain sensing for real-time structural health monitoring [58], while another study highlights their application in detecting degradation in polymer-based materials, further proving their versatility in sensing technologies [59]. While these materials have proven effective in these established applications, emerging research suggests potential extensions into areas such as flexible sensors, self-healing materials, and environmental monitoring devices.

On the other hand, various advanced fabrication techniques are employed to ensure uniform dispersion and effective integration of nanoparticles within the matrix, addressing the persistent challenge of achieving homogeneity in polymer nanocomposites. Efforts to enhance dispersion quality have focused on techniques that break up nanoparticle agglomerates and promote even distribution, thereby improving the functional surface area for interactions with target analytes [60]. Key techniques include ultrasonication, mechano-activation, toroidal stirring, and calendaring [61,62], which aid in breaking up nanoparticle agglomerates and promote even distribution, enhancing the functional surface area for interactions with target analytes. Sol–gel processing is another widely used method, as it enables precise control over the structure and porosity of the nanocomposites, which is essential for optimizing sensor performance [63]. Additionally, in situ polymerization allows for the direct formation of nanocomposites by polymerizing a monomer in the presence of dispersed nanoparticles, ensuring strong interfacial bonding and uniform distribution [64]. These fabrication techniques are pivotal in enhancing the stability, sensitivity, and selectivity of carbon-based nanocomposites, making them highly effective for advanced sensing applications. Beyond these advancements, the versatility of these fabrication methods has enabled the development of nanocomposites tailored for specific applications. These include flexible sensors for wearable technologies, self-healing materials for biomedical and structural health monitoring, and high-performance devices for environmental monitoring. As these techniques continue to evolve, they hold the potential to expand the functional capabilities of nanocomposites, making them suitable for increasingly complex and diverse sensing environments.

Figure 8, which shows the most frequent author’s keywords, mirrors the commented trends but provides additional specificity. Here, keywords such as “strain” and “3D printing” suggest active exploration into flexible, responsive materials for real-time monitoring, particularly in strain-sensitive applications. It is important to point out that in recent years, additive manufacturing technologies or 3D printing have emerged as transformative methods for fabricating nanocomposites with complex architectures and precise control over material distribution. This technique is especially valuable for creating strain sensors and other flexible, wearable devices that require precise structural configurations to respond to mechanical deformations. Furthermore, direct ink writing [65,66] and fused deposition modeling [67,68] are popular 3D printing techniques used for incorporating nanoparticles like graphene and carbon nanotubes into polymers, enhancing the electrical conductivity and mechanical strength of the printed structures. These additive manufacturing methods not only improve material efficiency and reduce waste but also open new possibilities for designing multifunctional sensors with complex geometries that were previously difficult to achieve using traditional fabrication methods [69]. Consequently, these technologies are becoming essential tools in the development of next-generation nanocomposite-based sensors, particularly for applications that require adaptability and high performance in real-time monitoring environments.

Additionally, keywords such as “wearable devices”, “environmental monitoring”, and “self-healing materials” align closely with emerging trends in the field. While these terms were not prominent in the bibliometric keyword frequency analysis, their growing significance is evident in recent studies exploring advanced sensor functionalities.

**Figure 9:**
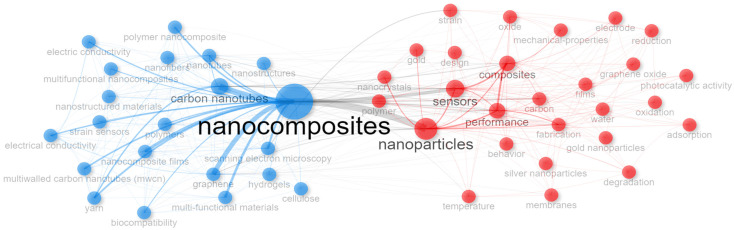
Keywords plus co-occurrence network.

The keywords co-occurrence network in Figure 9 reveals a thematic structure divided into two main clusters that represent key approaches in multifunctional nanocomposite research for sensing technologies. The central positioning of terms like “nanocomposites” and “nanoparticles” in the network serves as a bridge between both clusters, highlighting the interdependence between material innovation and application optimization. This scheme reflects the interdisciplinary nature of nanocomposite research, where advances in material properties directly translate into more robust and efficient sensing technologies.

On the left side (blue nodes), there is a focus on carbon-based materials as previously indicated. This cluster highlights the importance of terms like “strain sensors” and “electrical conductivity” that reflect the development of nanocomposite-based sensors that can detect changes in electrical resistance under strain, making them useful for applications in structural monitoring and wearable health devices. Keywords such as “hydrogels”, “cellulose”, “film”, and “biocompatibility” indicate interest in materials that can integrate with biological systems, which is crucial for wearable sensors in biomedical applications.

In the right cluster (red nodes), there is an emphasis on sensor applications and performance. This group suggests that researchers aim to optimize the functional properties of nanocomposites based on chemical parameters (e.g., “oxides”, “oxidation”, and “photocatalytic activity”), which are essential for sensor performance in diverse industrial and environmental settings [70,71]. The presence of keywords such as “graphene oxide”, “silver nanoparticles”, “gold”, and “nanocrystals” reflects the diversity of material approaches in developing sensors with high sensitivity and selectivity, with applications ranging from gas detection to pathogen monitoring. Additionally, terms like “membranes” and “films” indicate ongoing efforts in refining fabrication techniques that allow for the adequate structural and functional integration of nanocomposites into practical devices.

**Figure 10:**
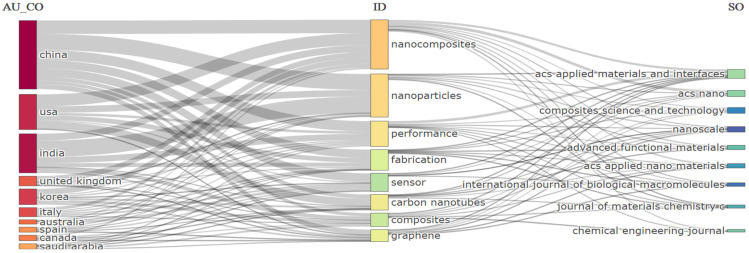
Sankey diagram with countries’, keywords’, and sources’ relationships.

The Sankey diagram in Figure 10 offers a comprehensive view of the interplay between countries (AU_CO), key research topics (ID), and publication sources (SO) in the field of multifunctional nanocomposites for sensing technologies. This visualization reveals that China and the USA are the leading contributors in this domain, indicating substantial research investment and output. Both countries show strong links to foundational topics such as “nanocomposites”, “nanoparticles”, and “carbon nanotubes”. This focus aligns with global trends, as these materials are foundational for enhancing sensor performance due to their mechanical strength, flexibility, and conductivity. On the topic of publication sources, the diagram reveals that research from leading countries is often channeled through high-impact journals such as *ACS Applied Materials and Interfaces*, *ACS Nano*, and *Advanced Functional Materials*. These journals are associated with critical keywords like “nanoparticles” and “fabrication”, which are essential in the development of multifunctional sensors. The focus on fabrication suggests that researchers are actively working to improve the production processes of nanocomposites, making them more scalable and functional for commercial sensing applications.

Notably, other countries, including India, the United Kingdom, and Korea, also make significant contributions to specific areas such as “graphene” and “carbon nanotubes”. These countries have emerging research outputs that align with their strengths in materials science and engineering. Furthermore, journals like *Composites Science and Technology* and *Chemical Engineering Journal* appear prominently, reflecting the interdisciplinary nature of this research field, where chemistry, material science, and engineering converge. This distribution of research output across different countries and journals underscores the collaborative and multifaceted approach necessary to advance nanocomposite-based sensing technologies on a global scale, with each country and journal contributing distinct expertise to the collective knowledge base.

**Figure 11:**
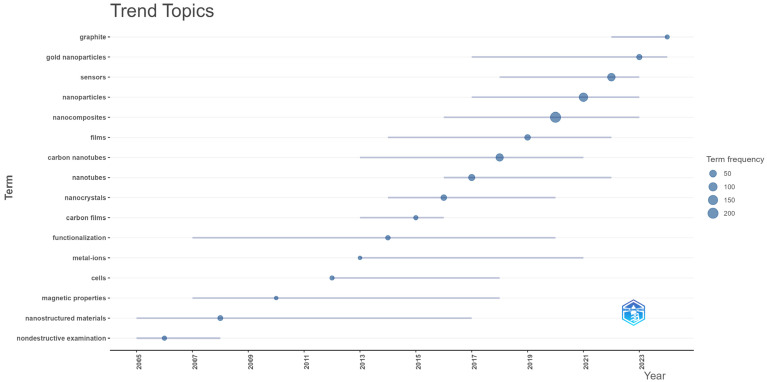
Trend topics based on keywords.

Figure 11 illustrates the evolution of research trends in nanocomposite materials for sensing applications from 2005 to 2023, highlighting the increasing complexity and specialization within the field. Early terms, such as “nondestructive examination” and “nanostructured materials” emerged around 2005, marking a foundational phase focused on the basic properties of nanomaterials for testing and diagnostics without damaging the material [72,73]. These initial investigations laid the groundwork for understanding how nanostructures could enhance sensing capabilities in non-invasive applications across various sectors, including construction, aerospace, and biomedicine. This period represents the field’s growing curiosity about nanomaterials and their potential to innovate material testing methods.

The trend line advances significantly post-2010, with terms such as “carbon nanotubes” and “nanocomposites” gaining frequency, reflecting a heightened interest in carbon-based materials due to their superior mechanical and electrical properties, essential for sensor technology enhancement. In recent years, terms like “graphene”, “gold nanoparticles”, and “sensors” themselves have become increasingly prominent. The rise of “graphene” around 2020 points to its versatile applications in flexible electronics and real-time sensing [74,75], while “gold nanoparticles” underscore developments in biochemical and environmental sensors, leveraging the material’s unique optical and conductive properties [76,77]. The increased frequency of “sensors” as a keyword in the latest years emphasizes a shift toward application-oriented research, focusing on optimizing nanocomposites for practical monitoring solutions.

**Figure 12:**
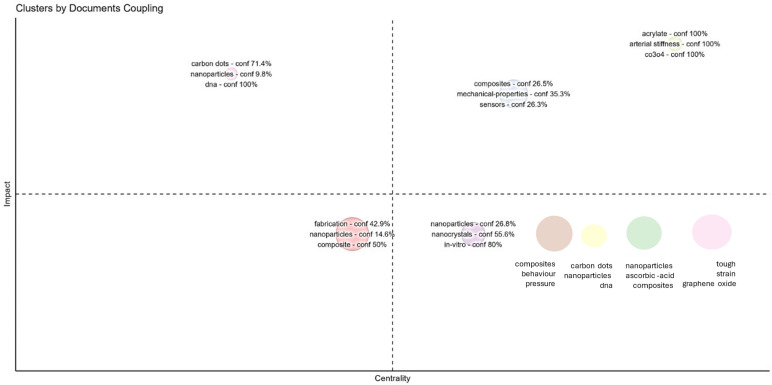
Conceptual structure map.

Figure 12 visualizes clusters based on document coupling, revealing central themes in multifunctional nanocomposites for sensing technologies by organizing research topics according to both impact (vertical axis) and centrality (horizontal axis). The clusters highlight distinct thematic areas. Cluster 1, positioned near the center, focuses on “fabrication”, “nanoparticles”, and “composites”, underscoring the fundamental role of material synthesis techniques in the broader nanocomposite research landscape. This central cluster represents foundational methods for creating nanocomposite structures, which are likely to support various specialized applications. Clusters positioned in the top-right quadrant, like Cluster 6 (“composites”, “mechanical-properties”, and “sensors”) and Cluster 7 (“tough”, “strain”, and “graphene oxide”), indicate high-impact, high-centrality topics, pointing to applied research on nanocomposites with specific mechanical and sensor-related properties. These clusters reflect a focus on enhancing durability, flexibility, and responsiveness—key attributes for sensing applications. Other clusters, such as Cluster 3 (“nanoparticles”, “nanocrystals”, and “in-vitro”) and Cluster 5 (“carbon dots”, “nanoparticles”, and “DNA”), showcase research with lower centrality but significant impact, suggesting niche yet influential studies, possibly in biomedical applications where specific functional properties, like biocompatibility and molecular detection, are crucial.

**Figure 13:**
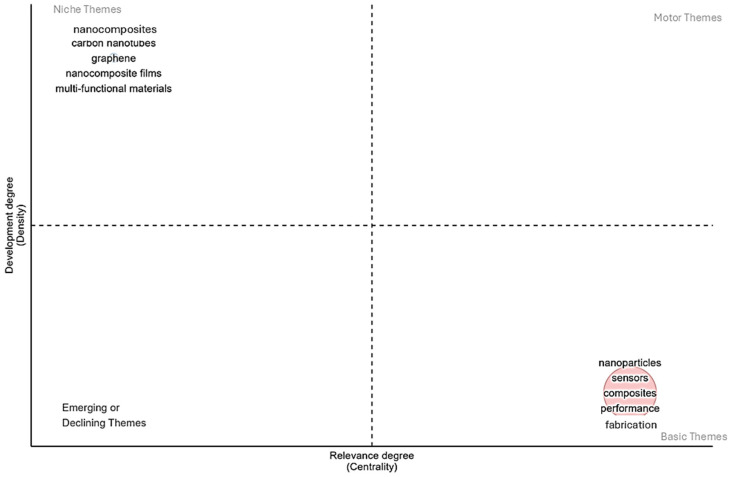
Thematic map of the topic.

Figure 13 illustrates the thematic landscape of nanocomposite research categorizing topics by development and relevance. Established themes like “nanocomposites”, “carbon nanotubes”, and “graphene” exhibit high development but are less integrated with other research areas indicating a focus on refining known material properties. Conversely, emerging themes such as “nanoparticles”, “sensors”, “composites”, and “fabrication” are highly relevant but less developed suggesting a growing interest in performance and application potential.

**Figure 14:**
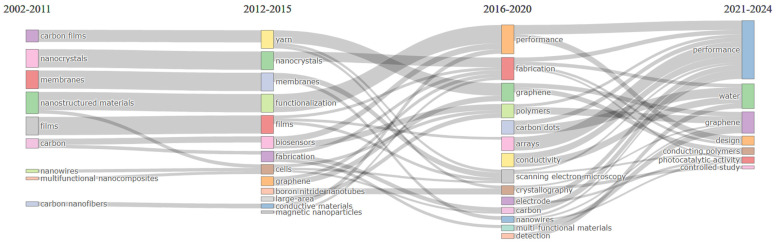
Thematic evolution of the topic.

Figure 14 demonstrates the thematic evolution of the research, revealing a progression from foundational topics to advanced applications and new materials. In the initial period (2002–2011), research centered on “carbon films”, “nanocrystals”, and “nanostructured materials” with an emphasis on the material properties of nanocomposites. From 2012 to 2015, themes such as “yarn”, “nanocrystals”, “functionalization”, “biosensors”, and “fabrication” gained prominence, reflecting an interest in expanding the applications of nanocomposites. Between 2016 and 2020, a significant shift emerged towards performance-focused topics such as “performance”, “fabrication”, “conductivity”, “carbon dots”, and “scanning electron microscopy”, indicating a deepened interest in optimizing and understanding material functionality. The use of scanning electron microscopy became prominent as it provided crucial insights into nanoparticle dispersion, facilitating more precise control and enhancement of nanocomposite properties. In the most recent period (2021–2024), research has shifted towards application-driven themes such as “conducting polymers”, “photocatalytic activity”, “water”, and “controlled study” reflecting a strong focus on leveraging nanocomposites for targeted technological advancements, with an emphasis on hydrogels. These themes underscore efforts to develop multifunctional materials with real-world applications, emphasizing the practical implementation of nanocomposites across diverse technological fields.

Multifunctional nanocomposites offer significant advantages for sensing technologies, particularly through the integration of advanced materials such as carbon nanocomposites, MXenes, and MOFs. Carbon nanocomposites, including carbon nanotubes and graphene, exhibit exceptional electrical conductivity, mechanical stability, and chemical durability, making them ideal for highly sensitive and robust sensors. Similarly, MOFs offer tunable porosity and chemical specificity, enabling efficient molecular recognition and improving sensor selectivity, while MXenes provide high electrical conductivity and tunable surface chemistry that improves material dispersion and interfacial interactions in polymeric matrices. Despite these strengths, several limitations remain. Scalability and cost-effectiveness remain significant challenges, as the manufacturing processes for many advanced nanocomposites are resource intensive. In addition, the limited biodegradability of some materials poses environmental concerns, particularly in applications requiring disposable sensors. The long-term stability and performance of these materials under various environmental conditions also needs to be further investigated.

The functionality of multifunctional nanocomposites is underpinned by robust theoretical frameworks. For example, percolation theory explains how the formation of conductive networks at critical nanoparticle concentrations enhances electrical conductivity, while surface chemistry effects elucidate the mechanisms driving sensitivity and selectivity in molecular detection. These theoretical principles not only guide the development of new materials but also inform the optimization of existing ones to meet the demands of next-generation sensing technologies. By addressing these strengths and limitations with a solid theoretical foundation, future research can advance the design and application of multifunctional nanocomposites in various fields, including biomedical diagnostics, environmental monitoring, and structural health assessment.

## 5. Conclusions

This bibliometric review underscores the growing interest in multifunctional nanocomposites for sensing applications, propelled by their unique combination of properties such as enhanced electrical conductivity, flexibility, and durability. Research has expanded significantly, moving from foundational studies to highly specialized applications, including wearable health monitors, environmental sensors, and industrial diagnostics. These advancements are marked by a trend towards complex, application-driven designs that leverage the materials’ ability to operate under dynamic and extreme conditions.

The most cited papers are grouped into three key areas in multifunctional sensing technologies: (i) porous materials and hybrid structures for enhanced conductivity and sensitivity, (ii) flexible and self-healing materials for real-time monitoring, and (iii) sustainable and biodegradable nanocomposites in sensor applications. Carbon-based nanocomposites, such as graphene or carbon nanotubes, play a pivotal role due to their remarkable properties, including exceptional electrical conductivity, mechanical strength, and high surface area. However, their performance is further enhanced through hybridization with materials like MXenes and metal–organic frameworks. MXenes provide higher electrical conductivity and tunable surface chemistry, facilitating better dispersion and stronger interfacial interactions within polymer matrices. Studies emphasize that combining different nanocomposites amplifies sensor performance, enabling real-time monitoring with increased accuracy. This focus on hybrid materials helps meet the demands for next-generation sensors in fields where precision and resilience are critical.

Key techniques such as ultrasonication, mechano-activation, toroidal stirring, and calendaring have greatly enhanced the dispersion of nanoparticles within the matrix, enabling the integration of the properties of the nanocomposites into sensing devices. Furthermore, additive manufacturing has emerged as a transformative process, allowing the precise fabrication of complex nanocomposite architectures with tailored material distribution for enhanced performance.

Future research directions should emphasize the development of materials with improved long-term stability and biodegradability for practical applications. This includes addressing challenges such as material degradation in harsh environments and enhancing compatibility with biological systems. Innovations in synthesis methods that enhance material performance while reducing production costs are critical. For example, leveraging green chemistry approaches or scalable manufacturing techniques can minimize environmental impact while maintaining efficiency. Additionally, exploring how these advancements can contribute to environmental sustainability by reducing waste and energy consumption during production is vital. Promoting broader adoption of nanocomposite-based technologies will require not only technical advancements but also interdisciplinary collaborations to integrate these materials into diverse real-world applications effectively.

While China leads in research output, this review identifies a low rate of international collaboration (3.1%), suggesting that increased global partnerships could foster innovation and diversity in research approaches. Future directions point toward customizable and sustainable nanocomposites, with advances in additive manufacturing enabling precise control over material structure. Additionally, biodegradable options like nanocellulose are emerging for disposable sensors, aligning with sustainability goals in healthcare and environmental monitoring. By integrating sustainable practices and expanding international cooperation, the field can better meet the evolving demands for advanced sensing solutions.

## Figures and Tables

**Table 1:** Annual scientific production and citations.

Year	MeanTCperArt	N	MeanTCperYear	Citable Years	Year	MeanTCperArt	N	MeanTCperYear	Citable Years
2002	40	1	1.74	23	2015	112.94	18	11.29	10
2005	0.75	4	0.04	20	2016	29.83	23	3.31	9
2006	74.17	6	3.9	19	2017	71.59	27	8.95	8
2007	34.67	6	1.93	18	2018	78.11	37	11.16	7
2008	24.8	5	1.46	17	2019	50.21	48	8.37	6
2009	69.33	6	4.33	16	2020	32.11	55	6.42	5
2010	75.82	11	5.05	15	2021	31.96	74	7.99	4
2011	81.33	6	5.81	14	2022	25.16	81	8.39	3
2012	48.38	13	3.72	13	2023	10.53	95	5.26	2
2013	53.24	17	4.44	12	2024	2.62	94	2.62	1
2014	103.71	17	9.43	11					

**Table 2:** Most impactful sources based on h-index and total citations.

Scientific Journal	h_Index	g_Index	m_Index	TC	NP	PY_Start
*ACS Applied Materials and Interfaces*	19	27	1.583	1974	27	2013
*ACS Nano*	12	15	0.8	3539	15	2010
*Advanced Functional Materials*	9	11	0.563	583	11	2009
*Nanoscale*	9	9	0.75	569	9	2013
*Composites Science and Technology*	8	9	0.615	381	9	2012
*Journal of Materials Chemistry C*	8	11	1	282	11	2017
*ACS Applied Nano Materials*	7	9	1.167	122	9	2019
*RSC Advances*	7	9	0.583	149	9	2013
*Chemical Engineering Journal*	6	14	1.2	285	14	2020
*Nanomaterials*	6	8	0.857	201	8	2018

**Table 3:** Most impactful sources based on Bradford’s law.

Scientific Journal	Rank	Freq	cumFreq	Cluster
*ACS Applied Materials and Interfaces*	1	27	27	Cluster 1
*ACS Nano*	2	15	42	Cluster 1
*Chemical Engineering Journal*	3	14	56	Cluster 1
*Advanced Functional Materials*	4	11	67	Cluster 1
*Journal of Materials Chemistry C*	5	11	78	Cluster 1
*ACS Applied Nano Materials*	6	9	87	Cluster 1
*Composites Science and Technology*	7	9	96	Cluster 1
*International Journal of Biological Macromolecules*	8	9	105	Cluster 1
*Nanoscale*	9	9	114	Cluster 1

**Table 4:** Countries that published the most over time.

Country	Articles	SCP	MCP	Freq	MCP_Ratio
China	236	234	2	0.366	0.008
India	92	87	5	0.143	0.054
United States	82	80	2	0.127	0.024
South Korea	35	34	1	0.054	0.029
Canada	16	16	0	0.025	0
Italy	16	16	0	0.025	0
United Kingdom	15	15	0	0.023	0
Spain	14	14	0	0.022	0
Iran	13	13	0	0.02	0
Pakistan	13	12	1	0.02	0.077

**Table 5:** Countries that received the most citations.

Country	TC	Average Document Citations
China	11,453	48.5
United States	2308	28.1
India	2028	22
Japan	1185	296.2
South Korea	1144	32.7
Canada	1017	63.6
Spain	703	50.2
France	456	65.1

**Table 6:** Most impactful documents based on the total number of citations received.

Reference	DOI	Total Citations	Total Citations per Year	Normalized Total Citations
[41]	10.1002/adma.201604898	771	96.38	10.77
[42]	10.1021/acsnano.8b05739	722	103.14	9.24
[43]	10.1021/acsnano.5b02781	708	70.8	6.27
[44]	10.1021/nn500722y	592	53.82	5.71
[45]	10.1038/asiamat.2010.32	503	33.53	6.63
[46]	10.1002/anie.202200705	470	156.67	18.68
[47]	10.1021/acsnano.5b01835	452	45.2	4
[48]	10.1021/acsami.8b20755	349	58.17	6.95
[49]	10.1021/acs.chemmater.7b01170	319	39.88	4.46
[50]	10.1021/acsnano.1c01751	317	79.25	9.92
[51]	10.1002/adma.201001410	317	22.64	3.9

**Table 7:** Comparative overview of most-important nanocomposites for sensing applications.

Nanocomposite	Key Properties	Application Areas	Advantages
Carbon-based (e.g., CNTs and Graphene)	High electrical conductivity, flexibility, chemical stability	Gas sensing, wearable devices, structural health monitoring	Cost-effective, scalable, and durable
Metal–Organic Frameworks (MOFs)	High porosity, tunable chemical functionality	Environmental monitoring, chemical sensing	Excellent molecular signal capture
MXenes	High conductivity, tunable surface chemistry	Biomedical sensing, electromagnetic shielding	Superior mechanical strength and stability
Hybrid (e.g., Graphene–MXene composites)	Synergistic properties, enhanced sensitivity	Multifunctional sensors for extreme environments	Combines benefits of individual components

## Data Availability

The original contributions presented in this study are included in this article; further inquiries can be directed to the corresponding authors.
